# A Long-Lasting Triamcinolone-Loaded Microneedle Patch for Prolonged Dermal Delivery

**DOI:** 10.5812/ijpr-138857

**Published:** 2024-02-27

**Authors:** Nasrin Zarei Chamgordani, Sasan Asiaei, Fatemeh Ghorbani-Bidkorpeh, Masoud Babaee Foroutan, Mostafa Dahmardehei, Hamid Reza Moghimi

**Affiliations:** 1 Department of Pharmaceutics and Pharmaceutical Nanotechnology, School of Pharmacy, Shahid Beheshti University of Medical Sciences, Tehran, Iran; 2 Sensors and Integrated Bio-MEMS/Microfluidics Laboratory, School of Mechanical Engineering, Iran University of Science and Technology, Tehran, Iran; 3 Department of Biomedical Engineering, AmirKabir University of Technology, Tehran, Iran; 4 Burn Research Center, Iran University of Medical Sciences, Tehran, Iran

**Keywords:** Microneedle, Triamcinolone, Solvent Casting, Skin Permeation, Drug Delivery, Scars

## Abstract

**Background:**

Scar is an unpleasant skin lesion that occurs following deep wounds or burns. The application of local triamcinolone is a common treatment for scar treatment and prevention, which should be repeated several times in conventional dosage forms. An effort has been made here to provide a prolonged triamcinolone dermal delivery by microneedle technology, which can also be used for wound closure.

**Objectives:**

This study aimed to develop a long-lasting polylactic acid (PLA) microneedle patch for the prolonged release of triamcinolone acetonide (TrA) that could potentially be used for closure of wound edges and scar prevention and treatment.

**Methods:**

In this study, 3% and 10% TrA-containing polymeric microneedles were fabricated using the micro molding-solvent casting method. Optical microscopy, X-ray diffraction analysis (XRD), Fourier-transform infrared spectroscopy (FT-IR), and differential scanning calorimetry (DSC) were used for the characterization of microneedles. Mechanical strength was evaluated using a compression test and methylene blue staining. Additionally, the insertion depth was determined by histopathological sectioning of human skin samples and also insertion into Parafilm^®^M as a skin model. The in vitro drug release profile of the microneedles was studied over 34 days, and the kinetic model was determined. The ex-vivo skin permeation of TrA was studied using a Franz-diffusion cell.

**Results:**

The TrA-containing PLA microneedles were fabricated with a uniform structure without any failure, deterioration, or loss of needles. Fourier-transform infrared spectroscopy and differential scanning calorimetry showed no interaction between TrA and PLA, and no effect on crystallinity and thermal behavior of TrA on polymer was detected. Microneedles showed appropriate mechanical properties, which were able to penetrate to about 900 - 1000 μm depth. Release profile from the whole body of 10% and 3% microneedle fitted to Higuchi model with cumulative amounts of 625 µg and 201.64 µg over 34 days. Release from the needles followed zero-order kinetic with cumulative amounts of 30.04 µg and 20.36 µg for 10% and 3%, respectively, for 34 days. Permeation was calculated to be 17 µg/day for 10% TrA-containing microneedle.

**Conclusions:**

The results suggested that suitable PLA microneedles containing TrA with prolonged release behavior can be successfully constructed with the solvent casting method.

## 1. Background

Scars are aberrant tissues that occur during the wound-healing process due to the excessive formation of the connective tissue as a result of continuous local inflammation and infection (1, 2). Hypertrophic scar and keloid are the main types of scars. Pain, itching, restriction in movement, and cosmetic aberrant are the major symptoms of scarring that severely affect the lives of patients and have physical, psychological, and social consequences (3, 4). Several therapeutics are effective in down-regulating scar formation, which is mainly related to their anti-inflammatory effect. Intralesional corticosteroids (5), fluorouracil (6), interferon (7) injections, light and laser therapy (8), and cryotherapy (9) are the most prevalent methods for scar management. Among these, intraregional corticosteroid injection is the most widely used approach (10). However, this encounters some limitations, such as pain and frequent administration for a long time with a need for injection by professional healthcare (10, 11). Scar prevention might be helpful during wound healing using conventional transdermal formulations (cream, lotion, gel, and ointment); however, this might not be effective for mature scars due to poor penetration.

Microneedle is a novel dermal or transdermal drug delivery system that physically bypasses physiological barriers. The effectiveness of microneedles in wound healing, tissue regeneration, and scar treatment has been confirmed in several studies (12, 13). Microneedles can be loaded with different therapeutic agents that promote wound healing and treat scars (14, 15). Moreover, microneedles act as mechanical stimulators for collagen deposition and reorganization (12) and reduce the stress of the microenvironment, which helps down-regulate scar formation (16).

In terms of drug delivery, microneedles have been categorized as hollow, coated, and dissolving. Due to safety issues, dissolvable microneedles, which are made of fast-dissolving polymers, have attracted the most attention. However, these microneedles release the cargo in a bolus; nevertheless, the polymer dissolves and cannot modulate the release kinetics (17). Recently, swellable and biodegradable microneedles have been designed for controlled release and long-time therapeutic transportation, which is especially interesting for the treatment of chronic cases (18, 19).

The present study aimed to develop a novel, long-lasting, controlled-release triamcinolone acetonide (TrA) microneedle based on polylactic acid (PLA) through a micro-molding solvent casting method. Polylactic acid is a biodegradable and biocompatible polymer widely used in biomedical applications, especially in controlled-release scopes (20). Triamcinolone acetonide is an anti-inflammatory drug that is widely used for scar treatment (21) and sometimes for the prevention of recurrence after scar excision surgery (21, 22).

The physicochemical properties of TrA-loaded controlled-release microneedles were characterized by optical microscopy, X-ray diffraction analysis (XRD), differential scanning calorimetry (DSC), and Fourier-transform infrared spectroscopy (FT-IR). Furthermore, mechanical properties were examined by evaluating the height reduction percentage after mechanical loading and methylene blue staining. The insertion depth of microneedles was evaluated by the histological sectioning of skin after microneedle treatment and quantified by Parafilm^®^M as a skin simulator. Afterward, TrA content and uniformity of its distribution, in-vitro release profile, and kinetic of release were determined. Ultimately, the ex-vivo study was conducted to determine TrA permeation through the skin.

## 2. Methods

### 2.1. Materials

Poly (lactic acid) (PLA) was obtained from Chemiekas GmbH (Austria). Triamcinolone acetonide (TrA) was purchased from Iran Daru Co. Dichloromethane (DCM), and high-performance liquid chromatography (HPLC) grade acetonitrile and methanol were obtained from Merck (Germany).

### 2.2. Microneedles Fabrication

**Figure 1.**
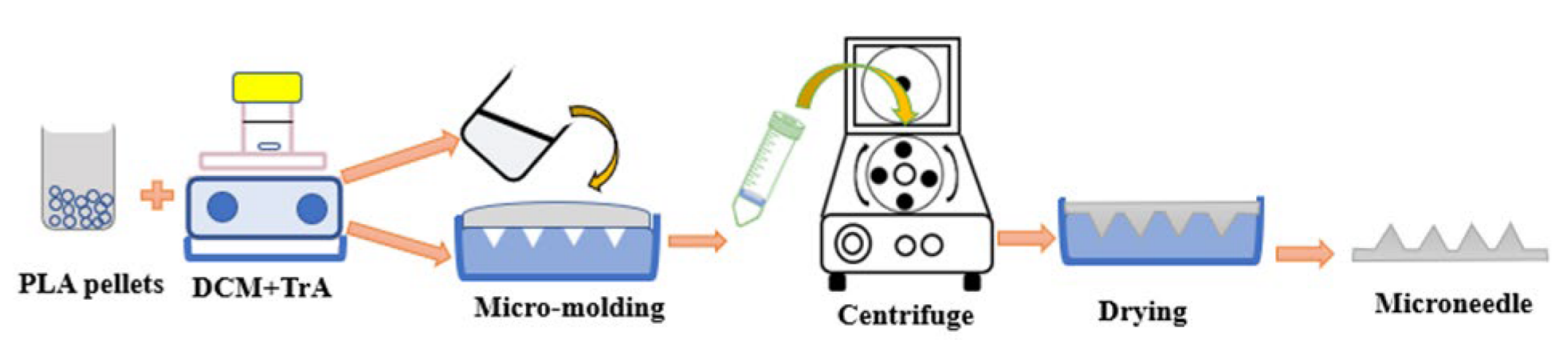
Schematic illustration of triamcinolone containing polylactic acid microneedle patch construction process

The fabrication of TrA/PLA microneedle patches is shown in Figure 1. Initially, 3% and 10% w/w (in polymer) of TrA solutions in DCM were prepared. Then, an appropriate amount of PLA was added and constantly stirred by magnetic stirring. Appropriate PLA content for the construction of microneedles with solvent casting method was previously developed and optimized to be 25% w/v (23). After complete dissolution, the polymeric solution was poured into a polydimethylsiloxane (PDMS) micro-mold and centrifuge (CELECTA LAB, TL220, China) at 4000 rpm for 40 minutes. Each mold had 81 square cross-section pyramidal shape with 1660 µm height, 24 µm tip diameter, and base width of 436 µm.

After drying for 24 hours at room temperature, the microneedles were removed from the molds and dried completely in a vacuum desiccator until reaching a constant weight. A microneedle patch without TrA was also fabricated and was used as a control.

### 2.3. Morphological Study of Microneedle Patch

The microneedles were visualized by a light microscope (Ceti, Medline Scientific, UK) and imaged with a digital camera (DFK MKU130, Source Imaging, Germany) to determine the geometrical parameters.

### 2.4. Fourier-Transform Infrared Spectroscopy (FT-IR)

Polylactic acid and triamcinolone acetonide interactions were studied using an ATR-FTIR spectrometer (Avatar 380, Thermo Nicolet, USA) in a region of 600 - 4000 cm^-1^ for plain, 10% TrA containing microneedle and TrA.

### 2.5. Differential Scanning Calorimetry

Microneedles were evaluated in terms of thermal and crystallinity behavior using differential scanning calorimetry (DSC) (DSC-60, Shimadzu, Japan) as described in a previous work (23). Briefly, the samples were heated (in an aluminum pan) from 0 to 200°C, then held at 200°C for 5 minutes, further cooled to 0°C and kept at this temperature for 5 minutes, and subsequently heated to 200°C. Each step was conducted at a 10°C/min rate under a nitrogen atmosphere with a 40 mL/min flow rate. Glass transition temperature (Tg), melting point (Tm), and degree of crystallinity ($\chi_{c}$) were calculated from the second heating scan. The percentage of crystallinity was determined by Equation 1, while w is the fraction of PLA in composite and $\Delta H_{m}$ is the melting enthalpy. The ${\Delta H^{0}}_{m}$ is the enthalpy of melting for 100% crystalline PLA and is determined to be 93.7J/g (24).

**Equation 1.**

%$\chi_{c}=\frac{∆H_{m}}{{w∆H^{0}}_{m}}\times 100$

### 2.6. X-ray Diffraction Analysis (XRD)

The crystalline state of TrA in fabricated microneedles was studied by X-ray diffraction ('X'Pert PRO MPD, PANalytical Company, The Netherlands) at 25°C with a Cu-Kα source at 40 mA and 40 KV; the samples were scanned at 2°-80°, and step time was 38 seconds.

### 2.7. Mechanical Properties of Microneedle Patches

The mechanical strength of the microneedle patches was studied by Brookfield texture analyzer (CT3, USA) in compression mode (25). Briefly, each microneedle was compressed between the fixed and moving parts of the instrument at a constant speed of 0.02 mm/s to reach a force of 32 N and held for 30 seconds. The height of the needles before and after compression was determined, and height reduction was calculated (26).

### 2.8. Insertion Ability of Microneedles

In-vitro insertion ability was conducted on full-thickness skin samples (female subjects aged 40 - 45 years), which were separated during cosmetic surgery. The underlying fatty layer was removed, and the samples were stored at -20°C until use. Skin samples were thawed at room temperature before experiments. Furthermore, TrA-loaded patches were manually pressed onto skin samples for 30 seconds. After removing the microneedle, the insertion site was immediately stained with 0.4% methylene blue solution. The excess stain was removed with an alcohol swab, and the punctured skin was photographed. To visualize the insertion depth, the inserted skin was fixed in 10% formalin, and histological sections were prepared and stained with hematoxylin and eosin (H&E) (27).

The validated Parafilm^®^M model, as a simulant of human skin, was used to intuitively investigate the depth of insertion. Ten layers of Parafilm^®^M were superimposed and placed on dental wax as support (28). Subsequently, the microneedle was applied manually for 30 seconds. After peeling off the microneedle, the number of holes in each layer of Parafilm^®^M was determined. The percentage of holes created in each layer was calculated by dividing the number of holes by the total number of needles. Considering a thickness of 127 µm for each layer, the insertion depth was calculated.

### 2.9. Drug Analysis

The concentration of TrA was determined using reverse-phase HPLC (Knauer, Germany) equipped with a stainless-steel column (RP18 column, 5 µm particle size, 250 mm × 4.6 mm i.d., PerfectSil Target) for separation and ultraviolet (UV) detector at 254 nm. The mobile phase was a mixture of 55:45 (v/v) water:acetonitrile, and the flow rate was adjusted to 1 mL/min (29). The limit of detection (LOD) and limit of quantification (LOQ) were measured to be 0.07 and 0.09 µg/mL, respectively.

### 2.10. Drug Content and Uniformity

To determine the TrA content, each microneedle patch (n = 3) was completely dissolved in DCM by sonication for 10 minutes. Methanol was then added to precipitate the polymer. Subsequently, the mixture was centrifuged at 12000 rpm for 10 minutes, and the supernatant was collected and analyzed by HPLC (30). Finally, the percentage of TrA recovery was calculated.

To study the uniformity of TrA distribution in an individual microneedle patch, each patch was divided into 3 pieces, and the percentage of TrA recovery in each part was determined as discussed.

### 2.11. In-vitro Drug Release and Kinetic Studies

To investigate the release from the whole body of the microneedle, each microneedle patch was placed in 50 mL of phosphate-buffered saline (PBS) containing 0.02% Tween 80 (31) and transferred to a shaking incubator (JTSDL40, JALTAJHIZ, Iran) (50 rpm) at 37 ± 1°C. At predetermined times until 72 hours, 1 mL of release medium was removed and immediately replaced with fresh buffer (at 37°C). The release medium was completely replaced with fresh buffer in other sampling times.

To study the release from needles alone, firstly, each microneedle patch was passed through a single layer of Parafilm^®^M, and the baseplate was sealed completely. Subsequently, each patch was floated on the surface of a 4 mL release medium while the needles were in contact with an aliquot (32). Release medium was sampled as previously described. Triamcinolone acetonide concentration in the sampling aliquots was analyzed using HPLC.

To study the mechanism of release of TrA from the whole body of microneedles and needles alone, the obtained release data were analyzed using zero-order, first-order, cylinder-type Fickian, and Higuchi mathematical models.

### 2.12. Ex-vivo Skin Permeation

Ex-vivo permeation experiments were performed on the full-thickness dorsal skin of male Sprague-Dawley rats. At first, the rats were anesthetized and sacrificed. Subsequently, the back region was trimmed and excised. Then, the adherent fat was removed, and the samples were stored in aluminum foil at -20°C until use. After thawing for 2 hours at room temperature, the microneedles were manually applied to the skin samples for 30 seconds. The samples were fixed between the donor and receptor compartments of Franz-diffusion cells. The receptor chamber was filled with 3 mL of buffer and placed at 37 ± 0.5°C with stirring. At each predetermined time, 1 mL of receptor medium was removed and replaced with the same volume of fresh buffer. The cumulative amount of permeated TrA was plotted as a function of time after HPLC analysis of TrA content. Permeation studies used microneedles and an O/W cream, all containing 10% TrA. The cream was formulated here and was used as a conventional dosage form just for comparison. All permeation experiments were conducted 3 times.

### 2.13. Statistical Analysis

The results are presented as mean ± standard deviation (SD). A *t*-test and one-way analysis of variance (ANOVA) with Tukey's post-hoc test were used for statistical comparisons, depending on the situation, using SPSS (Statistical Package for Social Science, IBM SPSS Statistics 22, New York, USA). A P-value less than 0.05 was considered statistically significant.

## 3. Results

### 3.1. Preparation of Microneedle Patch

**Figure 2.**
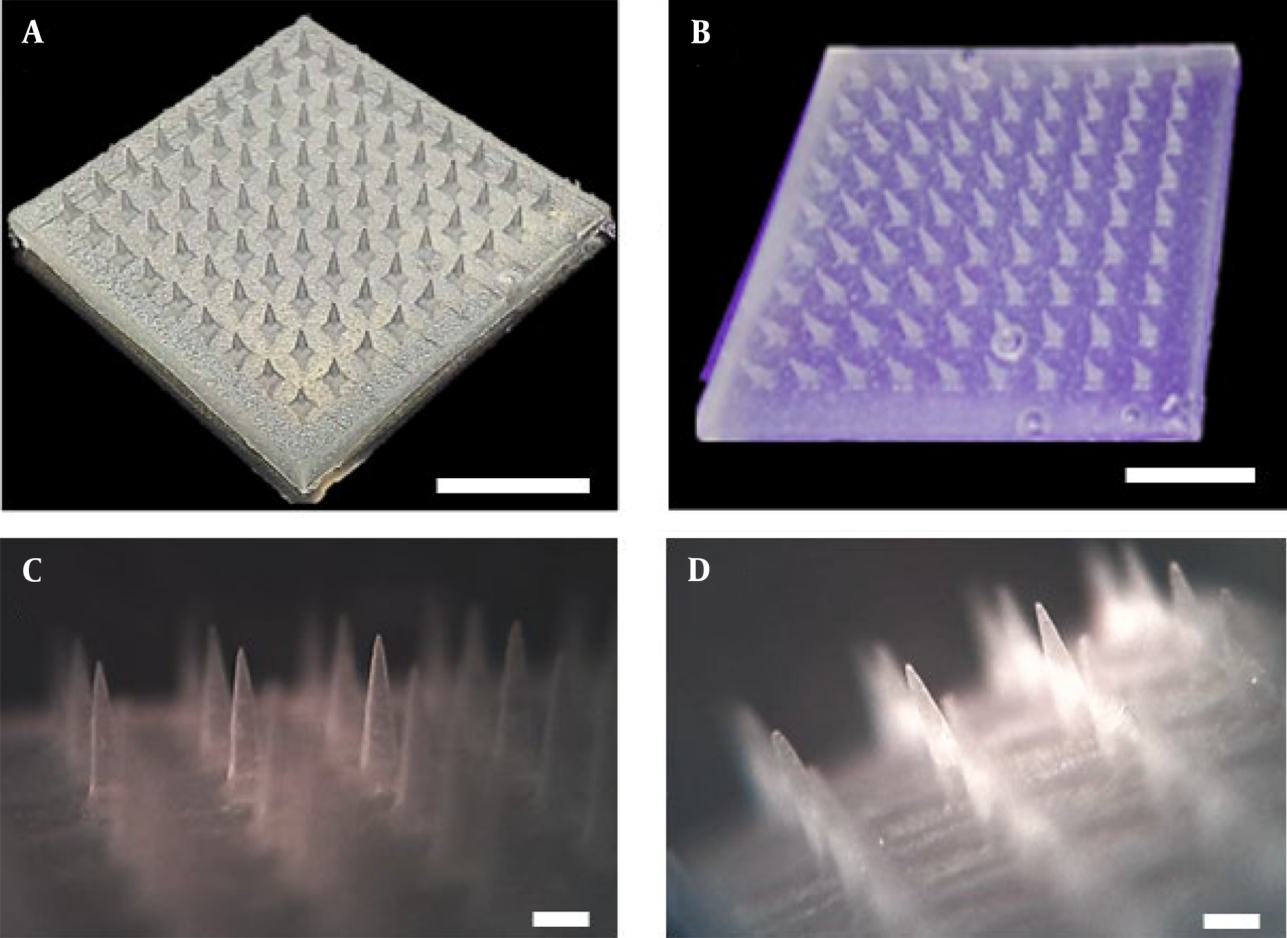
Photographic image of A, B, 10% and 3% TrA-containing microneedle (scale: 0.5 cm), respectively; Microscopic image of C, D, 10% and 3% TrA-containing microneedle (scale: 500 µm), respectively.

The 3% and 10% w/w TrA-containing PLA microneedles were fabricated using the solvent casting micro-molding method. As Figure 2A and B show, fabricated microneedles contain 81 (9 × 9) uniformly distributed needles without any failure, deterioration, or loss. Through microscopic analysis (Figure 2C and D), the microneedle dimensions were measured, and the results are presented in Table 1. The data show that there were no statistically significant differences between the geometry of different microneedles (P > 0.05).

**Table 1.** Dimension of Microneedles Measured by Optical Microscopy

Microneedle	Needle Height, µm	Base, µm	Needle-to-needle Distance, µm
**Plain**	1358 ± 54	418 ± 25	1408 ± 20
**3% TrA-containing**	1373 ± 62	424 ± 17	1414 ± 12
**10% TrA-containing**	1355 ± 50	428 ± 14	1410 ± 18

Abbreviation: TrA, triamcinolone acetonide.

### 3.2. Physicochemical Properties of Drug-Loaded Microneedles

#### 3.2.1. Fourier-Transform Infrared Spectroscopy

**Figure 3.**
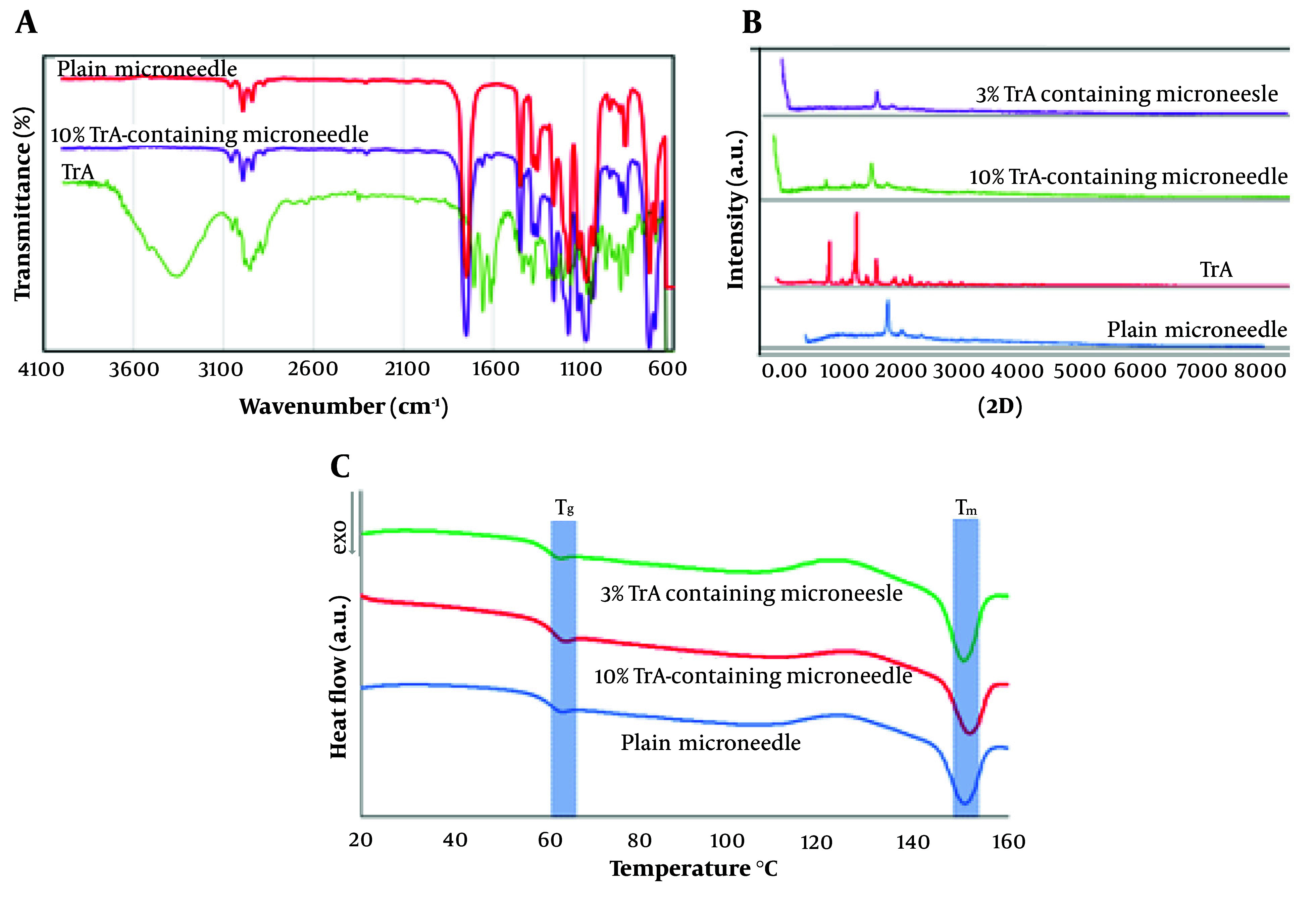
Physicochemical characterization of microneedles. A, FT-IR spectra of TrA powder, 10%-TrA containing and plain microneedle; B, XRD diffractogram of the plain, 3% and 10% TrA-containing microneedle; C, DSC thermograms of the plain, 10% and 3% TrA-containing microneedle.

Fourier-transform infrared spectroscopy (FT-IR) analysis of 10% TrA-containing and plain microneedle and TrA are presented in Figure 3A. The characteristic bands of the TrA spectrum were the broad peak at 3355 cm^-1^ (a typical absorption band for vibrational stretching of -O-H groups) and a peak at 1709 cm^-1^ (referred to as stretching vibration of the carbonyl group); these results follow the previous results (33).

The comparison of the FT-IR spectra for 10% TrA-containing and plain microneedles revealed no significant shifts in the main absorption bands of PLA (e.g., 1753 and 1456 cm^-1^, as discussed in a previous paper (23) in the formulations and no extra peaks were detected. However, the TrA bands were not disclosed, which might be masked by the PLA bands (possibly due to the lower concentration of TA). This experience has been confirmed in several studies (34). It can be concluded that there was no interaction between the PLA and TrA in this formulation.

#### 3.2.2. X-Ray Diffraction Analysis

X-ray diffraction analysis (XRD) patterns of 3% and 10% TrA-containing microneedle, plain microneedle, and TrA powder are shown in Figure 3B. Triamcinolone acetonide showed sharp diffraction peaks at 2θ values of 10.0°, 14.1°, and 17.1°, representing its high crystallinity. Microneedles containing 10% TrA represented characteristic diffraction peaks of TrA (e.g., 2θ = 9.9° and 14°), stating that the drug was dispersed as crystalline solid particles in the polymer matrix. The decrease in the intensity of the characteristic TrA diffraction peaks in this formulation, compared to TrA powder, could be attributed to the recrystallization after solvent evaporation in polymer matrices. Such behavior has also been expressed in various studies (35). However, in an X-ray diffractogram of 3%TrA containing microneedle, the characteristic peaks of TrA disappeared. This finding implies that the TrA was distributed in molecular form or was in an amorph state in the polymer matrix.

#### 3.2.3. Thermal Behavior

Differential scanning calorimetry thermograms of plain and TrA-loaded microneedles are presented in Figure 3C. Differential scanning calorimetry analysis was performed to investigate the effect of TrA content on the thermal behavior of the polymer and determine the crystallinity percentage of the polymer matrix.

The data showed that the addition of 3 and 10 wt% of TrA did not have any relevant changes in Tg and Tm. The crystallinity percentages of 10% and 3% TrA microneedles were calculated to be 14.49 ± 0.91 and 13.12 ± 2.03, respectively, which showed no statistically significant differences (P > 0.05). This finding indicates that increasing TrA content did not affect the crystallinity.

### 3.3. Mechanical and Insertion Properties

**Figure 4.**
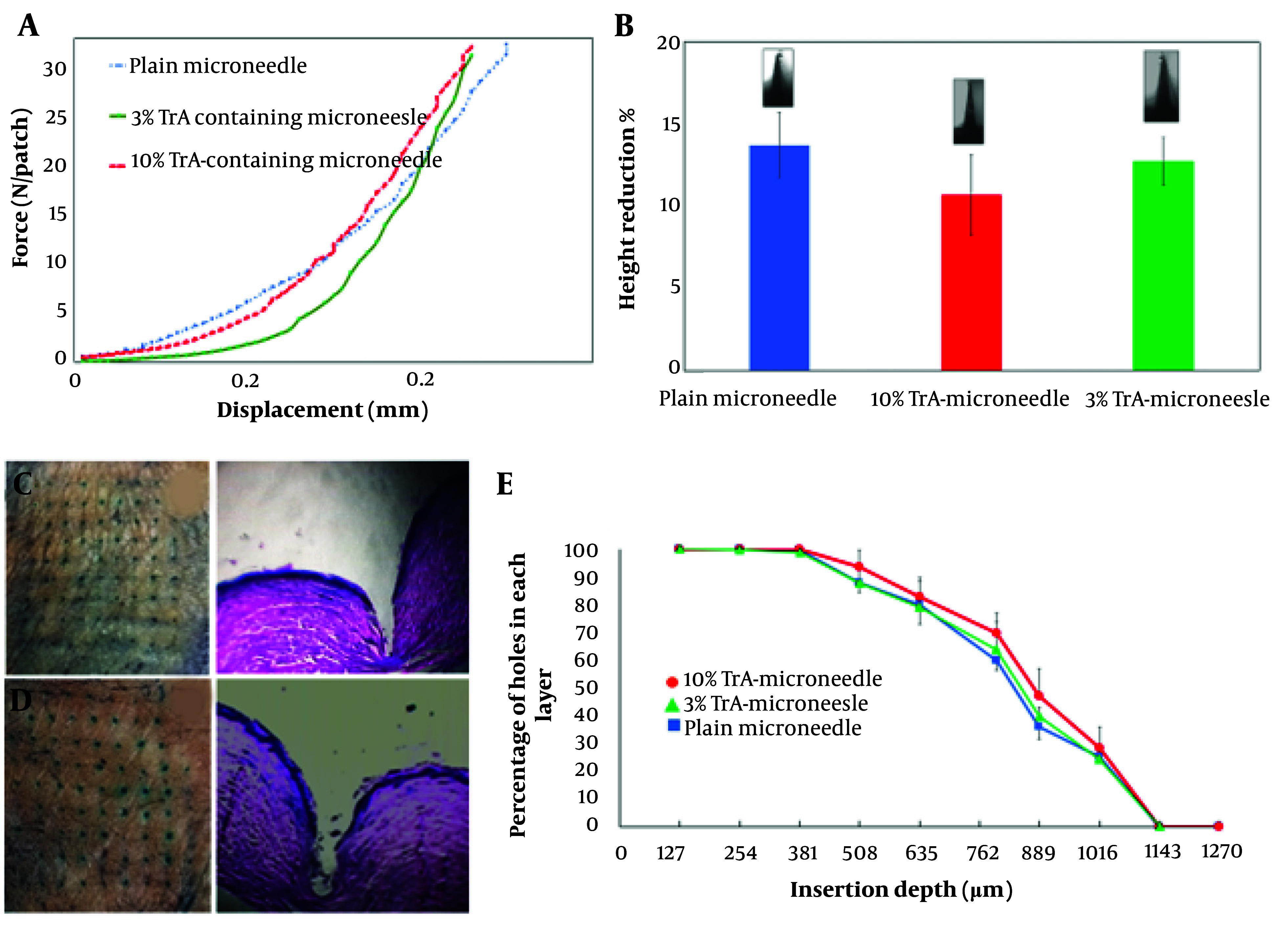
Mechanical strength and insertion properties of microneedles. A, Force-displacement curves for plain, 3% and 10% TrA-containing microneedles; B, Height reduction percentage of plain, 3% and 10%-TrA containing microneedles under 32 N compression force; C, D, 3% and 10% TrA-containing microneedle insertion into the human skin and corresponding pathological sectioning with H&E staining, respectively; E, percentage of holes created in each layer of Parafilm versus the insertion depth for plain, 3%, and 10%-TrA containing microneedles.

Figure 4A depicts force-displacement curves of different TrA-containing and plain microneedles, which show good mechanical strength without any failure under 32 N compression force.

The percentage of height reduction under 32N axial force for 10% and 3% TrA-containing microneedles were determined to be 10.72 ± 2.44 and 12.75 ± 1.42 (Figure 4B), respectively, which were not significantly different (P > 0.05).

The insertion capability of TrA-containing microneedles into the skin is presented in Figure 4C and D. For both microneedles (3% and 10%), penetration was confirmed by creating visible blue micro-holes (stained with methylene blue following microneedle insertion) in the excised human skin. Hematoxylin and eosin staining of tissue sections of microneedle-treated skin (Figure 4C and D) showed that 3% and 10% microneedles were penetrated to a depth of approximately 866 µm and 932 µm, respectively, which is approximately 64 - 69% microneedle length.

Figure 4E depicts the capability of insertion of TrA-containing and plain microneedles based on the percentage of holes created in each Parafilm^®^M layer. Both microneedles (3% and 10%) penetrated the seventh layer and reached the eighth layer (with a percentage of about 24 to 28%) of Parafilm^®^M. Therefore, the maximum depth obtained by the microneedles was 889 µm to 1016 µm, which closely followed the insertion depth calculated by the histological method.

### 3.4. Triamcinolone Acetonide Content and Uniformity

The average recovery percentage of loaded drug for three patches was determined to be 100.6 ± 1.49 and 95.18 ± 2.56 for 3% and 10% TrA-containing microneedles, respectively. In addition, the average recovery percentage for three parts of an individual patch containing 3% and 10% TrA were 104.10 ± 2.24 and 94.38 ± 0.9, respectively. High recovery percentage and low CV% showed complete and uniform distribution of TrA between patches and within each microneedle patch, indicating that the molding and centrifugation process did not affect drug distribution.

### 3.5. In-vitro Drug Release and Kinetics

**Figure 5.**
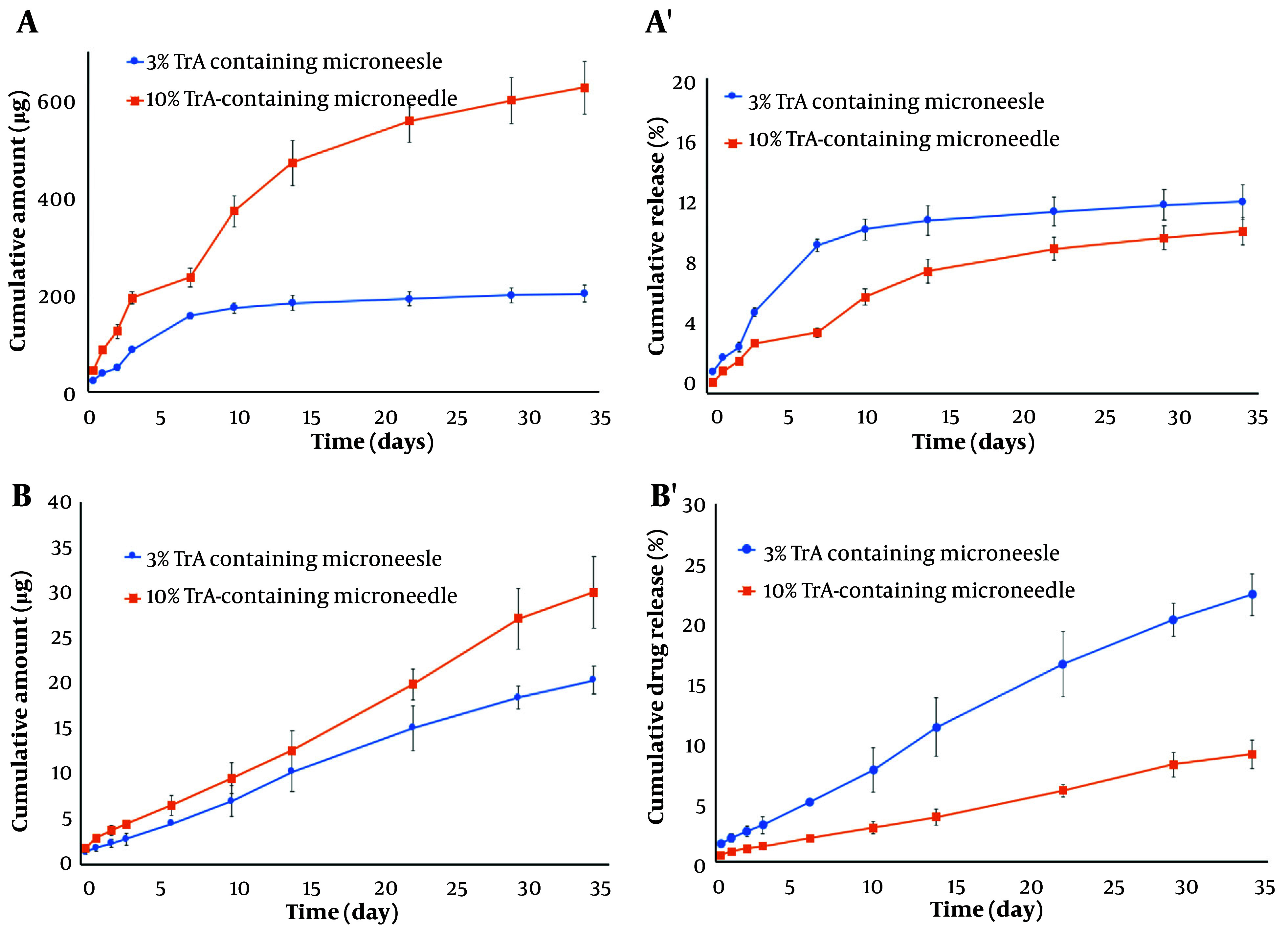
Profiles of release of TrA from the whole body (A and A') and needles only (B and B') of microneedles containing 3% and 10% TrA. A and B represent the cumulative amount released, and A' and B' represent the cumulative % released (mean ± SD, n = 3).

The release profile of TrA from the whole body of 3% and 10% TrA-containing microneedles is depicted in Figure 5A and A'. Following an ascending release in the first 7 days, a nearly linear release kinetic with the cumulative release of 625.78 ± 53.94 µg (10.31 ± 0.89%) and 201.64 ± 17.98 µg (12.19 ± 1.09%) for 10% and 3% formulation, respectively, were observed. Figure 5B and B' depicts the cumulative release of TrA from the needles of 3% and 10% TrA-containing microneedles. Cumulative release for 10% and 3% formulations were determined to be 30.05 ± 3.94 µg (9.00 ± 1.18%) and 20.37 ± 1.58 µg (22.38 ± 1.74%), respectively. Release data from the needles of two formulations of microneedle were described by zero-order kinetic (Table 2); however, the profile from the whole body was fitted to the Higuchi model (Table 3). 

**Table 2.** Fitting of Triamcinolone Release Data from Needles of 3% and 10% Triamcinolone Acetonide (TrA)-Containing Microneedles on the Different Kinetic Models with Correlation Coefficient (R^2^) and Release Rate Constant (k)

Formulation	Zero-Order		First-Order		Fickian-Cylinder	
	R^2^	K_0_ (µg. h^-1^)	R^2^	K_1_ × 10^-3^ (h^-1^)	R^2^	K_F_ (µg. h^-0.45^)
**3% TrA**	0.9946	0.0244	0.9028	2.309	0.9679	1.195
**10% TrA**	0.9967	0.0350	0.9416	2.764	0.9403	1.689

Abbreviation: TrA, triamcinolone acetonide.

**Table 3.** Fitting of Triamcinolone Release Data from Whole Body of 3% and 10% Triamcinolone Acetonide (TrA)-Containing Microneedles on Different Kinetic Models with Corresponding Correlation Coefficient (R^2^) and Release Rate Constant (k)

Formulation	Zero-Order		First-Order		Higuchi	
	R^2^	K_0_ (µg.h^-1^)	R^2^	K_1_ × 10^-3^ (h^-1^)	R^2^	K_H_ (µg.h^-0.5^)
**3% TrA**	0.7101	0.2310	0.5805	2.073	0.8715	7.351
**10% TrA**	0.9117	0.7158	0.7153	2.764	0.9796	24.11

Abbreviation: TrA, triamcinolone acetonide.

### 3.6. Ex-vivo Permeation Study

**Figure 6.**
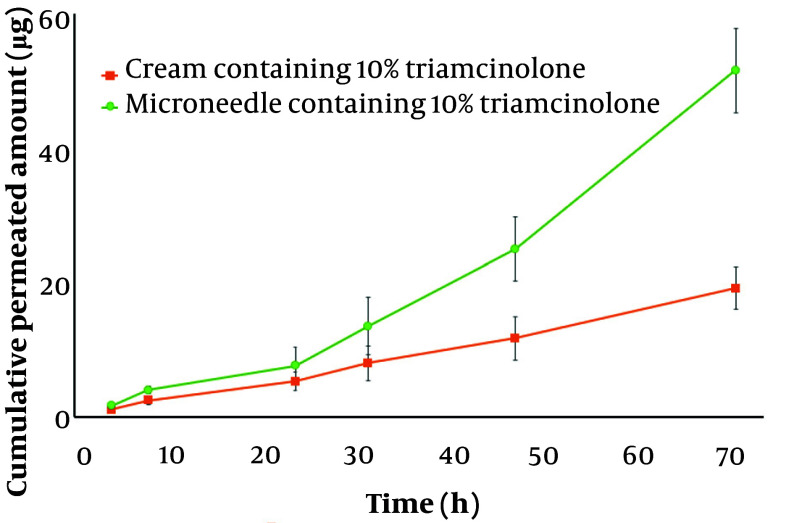
The cumulative amount of TrA (µg) permeated through rat skin from 10% TrA-containing microneedle and cream containing 10% TrA over 72 h (Mean ± SD, n = 3).

Based on the in-vitro results, since the microneedle with 10% TrA released more TrA during the test time, it was preferred as the optimal formulation for further ex-vivo experiments. The ex-vivo permeation profile of microneedle and cream containing 10% TrA are illustrated in Figure 6. After 72 hours, the cumulative amount of permeated TrA and permeation flux (the slop of permeation profile) was calculated to be 51.49 ± 6.28 µg and 0.91 ± 0.18 µg/h for microneedle and 19.43 ± 3.54 µg and 0.26 ± 0.05 µg/h for cream, respectively, which were statistically significant (P < 0.05). These data demonstrate the superiority of microneedles in skin delivery, compared to a conventional dosage form.

## 4. Discussion

Polylactic acid is a Food and Drug Administration (FDA)-approved polymer with biocompatibility and biodegradability properties, which has received special attention in pharmaceutical applications, especially for the controlled release of active ingredients. Recently, PLA has also been studied for the fabrication of microneedles. Different micro-molding techniques, including thermoforming and solvent casting, can be used for fabrication. Among these methods, solvent casting is preferred because it does not require harsh conditions (high temperature) and does not have other limitations (e.g., dependence on resolution, efficiency, and quality on operating parameters) of the thermoforming methods (36).

We previously developed the fabrication of PLA microneedles with a solvent casting method and optimized the polymer content to be 25% w/v (23). In the present study, the construction of 3% and 10% TrA containing microneedles is considered. Photographs and microscopic images showed the successful construction of microneedles. Additionally, a uniform distribution of TrA between microneedle patches (encapsulation of about 95% or more) and within an individual patch was observed for both formulations. Uniform distribution is essential to achieve controlled drug release. The uniform distribution of TrA into the PLA matrix was anticipated due to their compatibility and miscibility. The closeness of Hansen solubility parameters of TrA and PLA with a difference in total solubility (∆δt) equal to 3.7 MPa^1/2 ^(∆δt lower than 7 MPa^1/2 ^indicates the compatibility of drug and polymer) confirms the compatibility (37).

The mechanical properties of PLA depend on the crystallinity content (38). Differential scanning calorimetry was performed to determine the effect of TrA content on crystallinity, which showed no statistically significant effect between the crystallinity of TrA-containing microneedles. Moreover, FT-IR did not show the interaction between TrA and PLA at the maximum TrA content (10%).

The mechanical strength of the fabricated microneedles was confirmed in the compression test under 32 N (0.39 N/needle force), an average force of hand to manually insert the microneedle into the skin. The force-displacement profile did not show any failure (sudden force reduction during displacement); rather, microneedles became slightly compressed with height reduction. Studies have shown that the force required to insert a microneedle into the skin is less than 0.1 N per needle (39), suggesting that the TrA-containing microneedles and the plain microneedle can theoretically penetrate the skin without any failure.

The effective insertion of microneedles into the skin is an important parameter for efficient drug delivery. The number of micro-holes (which appeared as blue dots after methylene blue staining) created in the skin after using a microneedle can be a measure of the insertion capability of a microneedle (40). Both 3% and 10%-TrA loaded microneedles showed reasonable insertion ability into the excised skin with the creation of about 91% micro-holes in the skin (Figure 4D). Histological results support the effective insertion of microneedles into the skin.

Release from the whole body of the microneedle showed a typical logarithmic profile. This profile is created as a result of surface TrA dissolution and the creation of a drug depletion zone (41). As the thickness of the drug-free zone increases (thereby increasing the diffusion length), a slower release of TrA is observed over experiment time. Previous reports have shown similar behavior for the release of various drugs from the PLA matrix (39, 42). Release profiles from the whole body of microneedles were best fitted to the Higuchi model for both formulations (Table 3). This behavior was anticipated from the thin film (slab) geometry that was considered for the whole body of the microneedle.

For needles, the release profile from microneedles containing 3% and 10% TrA was well-fitted by a zero-order model. The difference in release behavior from the whole body of microneedles and needles alone can be attributed to different geometries. In monolithic systems (a system in which the drug is dissolved or dispersed in the polymer matrix), the geometry of the system has a significant effect on drug release (43). In these systems, the crystalline state of the loaded drug is another parameter that affects the drug release characteristics (44).

As the data show, there is a faster release (Figure 5) for the microneedle containing 3% TrA (less loading) than for the 10% TrA-containing microneedle for both releases from the whole body of the microneedle and needles alone. This finding might be attributed to the crystallin dispersion of TrA in 10% TrA-loaded microneedle compared to molecular or amorphous state dispersion of TrA in 3% TrA-loaded microneedle, as confirmed by XRD. As previous studies have shown, drug dispersion in crystalline form reduces the release rate compared to the amorphous state (45, 46). The cumulative permeated amount of TrA from TrA containing microneedle was about 3.5 times higher than TrA cream, which shows the superiority of microneedle in drug delivery over conventional drug delivery systems.

As mentioned before, the intralesional injection of TrA is the most widely used treatment for scars. Additionally, some clinical studies have reported successful treatment with no evidence of recurrence of keloid scars by the surgical excision of the lesion in conjunction with full-thickness skin grafting followed by the injection of TrA (47-49). However, the intralesional injection of TrA can lead to side effects, such as depigmentation/atrophy. These effects are hypothesized to be caused by the low solubility of TrA and the formation of microcrystals, which leads to the involution of subcutaneous fat lobules and the suppression of melanocyte function (50).

Using non-invasive transdermal delivery, such as microneedle, can reduce the side effects of intralesional injection. The excessive proliferation of fibroblasts is the main cause of scar formation. Studies show that when the concentration of TrA reaches 25 µg/mL, fibroblast viability starts to decrease, and when the concentration reaches 139 µg/mL, half of the maximum inhibitory effect is achieved (27). According to these data, it can be concluded that 10% TrA-containing microneedle with a cumulative permeation amount of 52 µg (in approximately 1 cm^3^) over 72 hours can have an inhibitory effect on fibroblasts. To date, various fast-dissolving TrA-containing microneedles have been developed for scar treatment. The superiority of the microneedle designed in the current study, compared to fast-dissolving microneedles, is its long-lasting characteristic, which can potentially be used for wound closure in addition to the inhibitory effect on fibroblast proliferation.

### 4.1. Conclusions

The present study demonstrated the construction of a long-lasting TrA-loaded PLA microneedle patch that can be used as a platform to deliver therapeutic agents in addition to potential use for wound closure. The microneedles were fabricated by the solvent casting method and showed reasonable mechanical strength. Microneedles were able to release their cargo for a long time. The authors believe that these microneedles could potentially be used for skin grafting after scar removal surgery to close the graft and as a TrA release platform to prevent scar recurrence.

## Data Availability

All required data are presented in the submitted manuscript as figures and tables. In addition, raw data are also available on request from the corresponding author during submission or after publication.
